# Mechanisms involved in reproductive damage caused by gossypol in rats and protective effects of vitamin E

**DOI:** 10.1186/s40659-015-0026-7

**Published:** 2015-07-31

**Authors:** Andréia T Santana, Marieli Guelfi, Hyllana C D Medeiros, Marco A Tavares, Paulo F V Bizerra, Fábio E Mingatto

**Affiliations:** Laboratory of Metabolic and Toxicological Biochemistry, UNESP-Univ Estadual Paulista, Rod. Comandante João Ribeiro de Barros (SP-294), km 651, Dracena, SP 17900-000 Brazil

**Keywords:** Gossypol, Fertility, Oxidative stress, Bioenergetics, Antioxidant system, Vitamin E

## Abstract

**Background:**

Gossypol is a chemical present in the seeds of cotton plants (*Gossypium* sp.) that reduces fertility in farm animals. Vitamin E is an antioxidant and may help to protect cells and tissues against the deleterious effects of free radicals. The aim of this study was to evaluate the mechanisms of reproductive toxicity of gossypol in rats and the protective effects of vitamin E. Forty Wistar rats were used, divided into four experimental groups (n = 10): DMSO/saline + corn oil; DMSO/saline + vitamin E; gossypol + corn oil; and gossypol + vitamin E.

**Results:**

Fertility was significantly reduced in male rats treated with gossypol in that a significant decrease in epididymal sperm count was observed (*P* < 0.05) and the number of offspring was significantly reduced in females mated with them (*P* < 0.05). This dysfunction was prevented by vitamin E. Gossypol caused a significant increase in the activity of the enzymes glutathione peroxidase (*P* < 0.01) and glutathione reductase (*P* < 0.01), but vitamin E did not reduce the enzyme activities (*P* > 0.05). The levels of reduced glutathione and pyridine nucleotides in testis homogenate were significantly reduced by gossypol (*P* < 0.05 and *P* < 0.01, respectively) and this reduction was accompanied by increased levels of oxidized glutathione (*P* < 0.05). Vitamin E showed a preventive effect on the changes in the levels of these substances. Gossypol significantly increased the levels of malondialdehyde (*P* < 0.01), a lipid peroxidation indicator, whereas treatment with vitamin E inhibited the action of the gossypol. Vitamin E prevented a decrease in mitochondrial ATP induced by gossypol (*P* < 0.05).

**Conclusions:**

This study suggests that the reproductive dysfunction caused by gossypol may be related to oxidative stress and mitochondrial bioenergetic damage and that treatment with vitamin E can prevent the infertility caused by the toxin.

## Background

Cottonseed meal is an agro-industrial co-product that can be used in animal feed. It is the second most important protein supplement available for animal feeding, exceeded only by soybean [1]. Although cotton meal is a lower-cost alternative, it has as an anti-nutritional factor, gossypol, a polyphenolic compound in the pigment-producing glands of the cotton seed. This substance causes toxicity and decreases fertility rates in ruminants and non-ruminants that ingest it in high concentrations or over a long period, since its effect is cumulative [2, 3].

Gossypol toxicity has been related to a decrease in antioxidant concentrations and increased formation of pro-oxidants, since it can interact with biological membranes by promoting the formation of reactive oxygen species. In high doses, it reduces the activity of enzymatic systems of the mitochondrial electron transport chain and disconnects respiration and oxidative phosphorylation, presenting an uncoupling effect [4]. Deleterious effects of gossypol on fertility have been reported in the literature. Among the negative aspects observed in several species are damage to the germinal epithelium, causing a decrease in spermatogenesis; and decreased motility and number of spermatozoa as a result of degeneration of testicular tissue, which implies a decrease in the number of sperm that would reach maturity and increased percentage of abnormal sperm [5–10].

Vitamin E is a fat-soluble vitamin present in biological membranes, which has an antioxidant role and can contribute to the protection of cells and tissues against the deleterious effects of free radicals. It has also been shown to increase sperm concentration in the ejaculate [11, 12]. This study aimed to evaluate the effects of gossypol on fertility in rats, including the assessment of oxidative damage and mitochondrial bioenergetics and analysis of the protective action of vitamin E.

## Methods

### Treatment of animals

The experimental protocols were approved by the Ethical Committee for the Use of Laboratory Animals of the UNESP—Univ Estadual Paulista, Campus de Dracena, SP, Brazil (Protocol number 19/2012). Male Wistar rats weighing approximately 200 g were used in this study. The animals were obtained from the Central Bioterium of UNESP—Univ Estadual Paulista, Campus de Botucatu, SP, Brazil, and were maintained with a maximum of four rats per cage under standard laboratory conditions with water and food provided ad libitum.

The rats were randomly divided into four groups of ten animals each, according to the following treatments: Group 1 (G1) received corn oil by gastric gavage and a mixture of dimethyl sulfoxide (DMSO) and saline (0.9% NaCl) i.p.; Group 2 (G2) received vitamin E (100 mg/kg BW) dissolved in corn oil by gastric gavage and a mixture of DMSO and 0.9% NaCl i.p.; Group 3 (G3) received Gossypol Acetic Acid (5 mg/kg BW) dissolved in a mixture of DMSO and 0.9% NaCl i.p. and corn oil by gastric gavage and Group 4 (G4) received Gossypol Acetic Acid (5 mg/kg BW) dissolved in a mixture of DMSO and 0.9% NaCl i.p. and vitamin E (100 mg/kg BW) dissolved in corn oil by gastric gavage. Gossypol and vitamin E dose selection was based on previous reports [13, 14].

### Fertility test

Fertility was estimated in four male rats of each group. After 14 days of treatment, each male was placed in an individual cage with two virgin untreated females of the same strain. They were left together for 10 days during which two estrus cycles should have elapsed [15]. After this period, the male rats were removed and the females kept in separate boxes until delivery. Once the rats calved, the quantity and the weight of offspring in each group were assessed.

### Sperm counting

After 14 days of treatment six of the animals of each group were euthanized by decapitation and the testes and epididymides were collected.

The tail of the epididymides, previously cut into small pieces with scissors, was used for semen collection and the subsequent counting of sperm.

For the analysis of the total number of epididymal sperm, the epididymal tail of each animal was placed in 10 mL of normal saline (0.9% NaCl) and homogenized under cooling. One hundred microliters of the resulting mash of each epididymis was placed in an individual “eppendorf” type tube and 900 µL of 0.9% NaCl was added to a final volume of 1 mL. The number of sperm in this obtained solution was counted in 128 small squares of a Neubauer chamber. Counting was performed in an optical microscope with 40× magnification. The number of spermatozoa was determined using the following formula:

$${\text{S}} = {\text{C }} \times {\text{ V }} \times {\text{ CF}}$$

where S = Sum total per animal; C = number of counted spermatozoa; FC = factor of the camera (1.25) and V = dilution (10^6^).

### Homogenate preparation

The tunica albuginea and the main vessels were removed and each testis was placed in 25 mL of medium containing 250 mM sucrose, 0.2 mM EGTA, 0.1 mM EDTA, 5 mM HEPES–KOH (pH 7.4) and 0.1% bovine serum albumin (BSA), maintained at 4°C and then sliced and homogenized with a Potter-Elvehjem homogenizer. The protein concentration of the homogenate was determined using the biuret reaction with BSA as a standard [16].

### Glutathione peroxidase activity

The activity of glutathione peroxidase (GPx) was determined by an indirect method based on the oxidation of GSH to GSSG, with the consequent oxidation of NADPH catalyzed by glutathione peroxidase [17]. One milliliter of 0.1 mM sodium phosphate buffer, pH 7.6, with 0.5 mM EDTA, 10 µL of 10% Triton X-100, testis homogenate (1 mg of protein) and 10 µL of 100 mM GSH and 10 µL of 25 mM H_2_O_2_ were added to 4 mL quartz cuvettes. After incubating the samples at 30°C for 5 min, 10 µL of 20 mM NADPH was added, and the variation in absorbance was determined at a wavelength of 340 nm in a spectrophotometer (Beckman-Coulter model DU-800, Fullerton, CA, USA). The oxidation of 1 µmol NADPH/min was used as a unit of GR. The specific activity was expressed as unit per mg of protein.

### Glutathione reductase activity

The activity of glutathione reductase (GR) was determined based in the reduction of GSSG to GSH by monitoring the oxidation of NADPH [18]. One milliliter of 0.1 mM sodium phosphate buffer, pH 7.6, with 0.5 mM EDTA, 10 µL of 10% Triton X-100, testis homogenate (1 mg of protein) and 10 µL of 100 mM GSSG were added to 4 mL quartz cuvettes. After incubating the samples at 30°C for 5 min, 10 µL of 10 mM NADPH was added, and the variation in absorbance was determined at a wavelength of 340 nm in a spectrophotometer (Beckman-Coulter model DU-800, Fullerton, CA, USA). The oxidation of 1 µmol NADPH/min was used as a unit of GR. The specific activity was expressed as unit per mg of protein.

### Glutathione assay

The levels of GSH and GSSG were determined by a fluorometric reaction with *o*-phthalaldialdehyde (OPT) [19]. Testis homogenate (1 mg of protein) was added to medium (125 mM sucrose, 65 mM KCl and 10 mM HEPES–KOH, pH 7.4) to a final volume of 1 mL and treated with 0.5 mL of 13% trichloroacetic acid. The mixture was stirred and then centrifuged at 9,000×*g* for 3 min. For GSH levels, aliquots (100 µL) of the supernatant were mixed with 2 mL of 100 mM NaH_2_PO_4_ buffer at pH 8.0 containing 5 mM EGTA. One hundred microliters of a OPT solution (1 mg/mL) was added, and the fluorescence was measured 15 min later in a spectrofluorometer (Shimadzu-RFPC 5301, Tokyo, Japan) using 350/420 nm as the excitation/emission wavelength pair.

For GSSG levels, the supernatant was treated with 20 mM *N*-ethylmaleimide, which reacts with free thiol groups. Aliquots (100 µL) of samples were mixed with 1 mL of 1 M NaOH followed by OPT. The data are expressed in nmol/mg protein estimated using a standard curve.

### Determination of NADPH level

Testis homogenate (2.0 mg protein) was added to medium (125 mM sucrose, 65 mM KCl and 10 mM HEPES–KOH, pH 7.4) to a final volume of 2.0 mL and centrifuged at 8,000×*g* for 3 min. The supernatant was collected, and the fluorescence was measured in a spectrofluorometer (Shimadzu-RFPC 5301, Tokyo, Japan) using 366/450 nm as the excitation/emission wavelength pair. The data are expressed in relative fluorescence units.

### Membrane lipid peroxidation (LPO) assay

The level of LPO was estimated by malondialdehyde (MDA) generation [20]. The testis homogenate (5 mg of protein) was added to a tube. Following the addition of 0.2 mL of 8.1% SDS, 1.5 mL of 20% acetic acid and 1.5 mL of 0.67% thiobarbituric acid (TBA, aqueous solution), glass-distilled deionized water was added to a final volume of 4 mL. The mixture was incubated for 60 min at 85°C. The MDA-TBA complex was extracted with 5 mL of n-butanol and the absorbance was measured at 535 nm in a Genesys 10 UV spectrophotometer (Thermo Spectronic, Rochester, NY, USA). The MDA concentration was calculated with ε = 1.56 × 10^5^/M/cm.

### Isolation of testicular mitochondria

Testis mitochondria were prepared according to the methodology of Amaral et al. [21]. Part of the homogenate was centrifuged at 2,500×*g* for 10 min, and the supernatant fluid centrifuged at 10,000×*g* for 10 min. The pellet (mitochondrial fraction) was resuspended and repelleted twice at 10,000×*g* for 10 min. EGTA, EDTA and defatted BSA were omitted from the washing medium. Mitochondrial protein content was determined by the biuret method.

### ATP quantification

ATP levels were determined using the firefly luciferin–luciferase assay system [22]. The mitochondrial suspension (1 mg protein) was suspended in 1 mL of a medium containing 65 mM KCl, 125 mM sucrose and 10 mM HEPES–KOH, pH 7.2 and centrifuged at 12,000×*g* for 10 min at 4°C, and the pellet was treated with 1 mL ice-cold 1 M HClO_4_. After centrifugation at 12,000×*g* for 10 min at 4°C, 100 µL aliquots of the supernatants were neutralized with 5 M KOH, suspended in 100 mM TRIS–HCl, pH 7.8 (1 mL final volume), and centrifuged at 12,000×*g* for 10 min. The supernatant was processed with a Sigma/Aldrich assay kit (Catalog Number FLAA) according to the manufacturer’s instructions and measured using a SIRIUS luminometer (Berthold, Pforzheim, Germany).

### Statistical analysis

Significant differences were calculated by one-way analysis of variance (ANOVA) followed by the Tukey test using the GraphPad Prism software, version 4.0 for Windows (GraphPad Software, San Diego, CA, USA). Values of *P* < 0.05 were considered significant.

## Results

### Effect of gossypol on fertility and body weight of offspring

Table 1 shows the number of males used for crossing per group, the number and percentage of females that were fertilized, the total number and the average weight of the offspring. A beneficial effect of vitamin E on fertility can be observed, since all females of group (G2) were fertilized and also had the largest number of offspring. The gossypol presented an adverse effect on fertility, because no female was fertilized (G3) and the number of offspring of this group was zero. In G4, where the animals were treated with gossypol plus vitamin E, 50% of females were fertilized and the number of offspring was close to that of the control group. The weight of offspring at birth was significantly different among all groups.

**Table 1:** Effect of gossypol on the fertility of male rats and weight of offspring and protective action of vitamin E

Treatment	Number of males	Number (%) of pregnant females	Number of offspring	Weight of offspring^d^, g
G1	4	8/7 (87.5)	65^ab^	6.54 ± 0.85ª
G2	4	8/8 (100)	89^a^	5.98 ± 0.60^b^
G3	4	8/0 (0)	0^c^	–
G4	4	8/4 (50)	42^b^	5.44 ± 0.53^c^

^a,b,c^Values in the same column with different superscripts differ (*P* < 0.05).

^d^Results expressed as mean ± standard deviation.

### Effect of gossypol on the epididymal sperm count

**Fig. 1:**
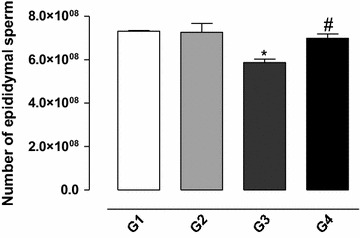
Number of sperm in the epididymis of rats exposed to gossypol and the protective action of vitamin E. The results represent the mean ± SEM of six animals per group. G1 = control; G2 = vitamin E 100 mg/kg BW; G3 = gossypol 5 mg/kg BW; G4 = gossypol 5 mg/kg BW + vitamin E 100 mg/kg BW. *Significantly different from control (G1) (*P* < 0.05). ^#^Significantly different from the group treated with gossypol (G3) (*P* < 0.05).

The treatment with gossypol 5 mg/kg BW (G3) decreased the sperm count significantly in the epididymis tail in comparison with the control group (G1) (*P* < 0.05) (see Fig. 1). The simultaneous treatment with gossypol and vitamin E 100 mg/kg BW resulted in counts similar to control values (G4).

### Effect of gossypol on glutathione peroxidase and glutathione reductase activity

**Fig. 2:**
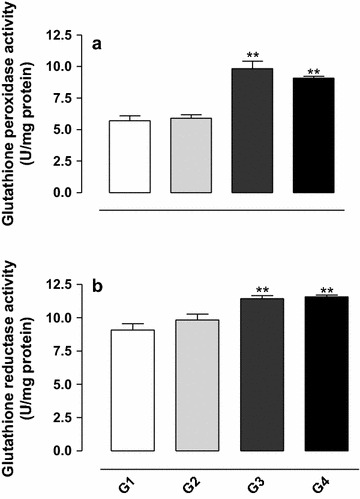
Activity of the enzymes glutathione peroxidase (**a**) and glutathione reductase (**b**) in testis homogenate from rats exposed to gossypol and the protective action of vitamin E. The results represent the mean ± SEM of six animals per group. G1 = control; G2 = vitamin E 100 mg/kg BW; G3 = gossypol 5 mg/kg BW; G4 = gossypol 5 mg/kg BW + vitamin E 100 mg/kg BW. **Significantly different from control (G1) (*P* < 0.01).

The glutathione peroxidase activity was significantly increased in the group treated with gossypol (G3) compared with the control (G1) (*P* < 0.01) (see Fig. 2a), and the simultaneous treatment of the animals with gossypol and vitamin E (G4) did not prevent the increase in glutathione peroxidase activity (*P* < 0.01).

Similar results were observed for glutathione reductase activity (see Fig. 2b).

### Effect of gossypol on the oxidative state of glutathione

**Fig. 3:**
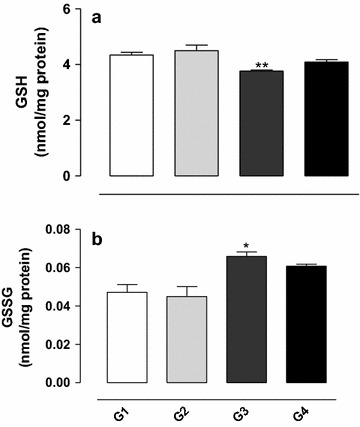
Concentration of reduced (**a**) and oxidized (**b**) glutathione in testis homogenate from rats exposed to gossypol and the protective action of vitamin E. The results represent the mean ± SEM of six animals per group. G1 = control; G2 = vitamin E 100 mg/kg BW; G3 = gossypol 5 mg/kg BW; G4 = gossypol 5 mg/kg BW + vitamin E 100 mg/kg BW. *^,^**Significantly different from control (G1) (*P* < 0.05 and *P* < 0.01, respectively).

Administration of gossypol (G3) induced a significant reduction in GSH concentration in the testis homogenate (*P* < 0.01) (see Fig. 3a). The simultaneous treatment with gossypol and vitamin E (G4) resulted in concentrations similar to control values.

A significant increase in the concentration of GSSG was observed in the testis homogenate of animals treated with gossypol (G3) (*P* < 0.05), indicating that this substance induced the oxidation of glutathione present in the homogenate (see Fig. 3b). The simultaneous administration of gossypol and vitamin E (G4) had a protective effect on this oxidation.

### Effect of gossypol on the oxidative state of pyridine nucleotides

**Fig. 4:**
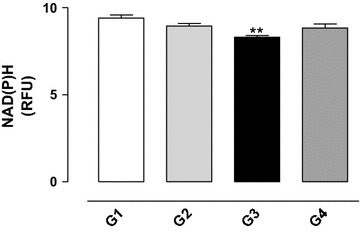
Level of pyridine nucleotides (NADP(P)H) in the testis homogenate from rats exposed to gossypol and the protective action of vitamin E. The results represent the mean ± SEM of six animals per group. G1 = control; G2 = vitamin E 100 mg/kg BW; G3 = gossypol 5 mg/kg BW; G4 = gossypol 5 mg/kg BW + vitamin E 100 mg/kg BW. **Significantly different from control (G1) (*P* < 0.01). *RFU* relative fluorescence units.

A significant reduction in the NAD(P)H concentration in the testis homogenate of the gossypol treated group (G3) was observed (*P* < 0.01) (see Fig. 4), showing that gossypol promoted the oxidation of pyridine nucleotides. The simultaneous treatment with gossypol and vitamin E (G4) resulted in concentrations similar to control values.

### Effect of gossypol on membrane lipid peroxidation

**Fig. 5:**
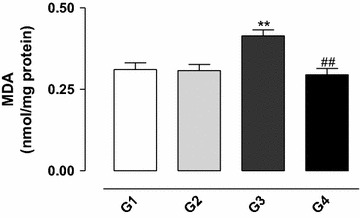
Concentration of malondialdehyde (MDA) in the testis homogenate from rats exposed to gossypol and the protective action of vitamin E. The results represent the mean ± SEM of six animals per group. G1 = control; G2 = vitamin E 100 mg/kg BW; G3 = gossypol 5 mg/kg BW; G4 = gossypol 5 mg/kg BW + vitamin E 100 mg/kg BW. **Significantly different from control (G1) (*P* < 0.01). ^##^Significantly different from the group treated with gossypol (G3) (*P* < 0.01).

The peroxidation of membrane lipids was assessed by the measurement of malondialdehyde (MDA). The results demonstrated that treatment with gossypol (G3) significantly increased the concentration of MDA (*P* < 0.01) (see Fig. 5), and the simultaneous treatment of the animals with gossypol and vitamin E (G4) significantly reduced the production of MDA (*P* < 0.01), indicating a protection against the harmful effects of gossypol on the lipids.

### Effect of gossypol on the level of ATP in testicular mitochondria

**Fig. 6:**
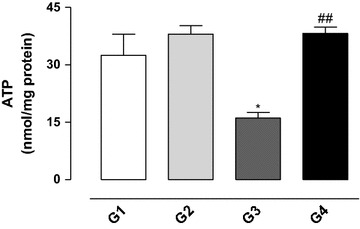
Concentration of ATP in the testis mitochondria from rats exposed to gossypol and the protective action of vitamin E. The results represent the mean ± SEM of six animals per group. G1 = control; G2 = vitamin E 100 mg/kg BW; G3 = gossypol 5 mg/kg BW; G4 = gossypol 5 mg/kg BW + vitamin E 100 mg/kg BW. *Significantly different from control (G1) (*P* < 0.05). ^##^Significantly different from the group treated with gossypol (G3) (*P* < 0.01).

Gossypol caused a significant inhibition of ATP synthesis in the mitochondria isolated from the rat testis while vitamin E resulted in ATP concentrations similar to the values for the control group (see Fig. 6).

## Discussion and conclusion

Deleterious effects of gossypol on fertility have been widely reported in the literature. Reduction in sperm concentration, inhibition of sperm motility and increased sperm mortality are among the weaknesses observed in various species [5–10, 13, 23–25]. In this study, treatment with gossypol 5 mg/kg BW resulted in a reduction in the total number of sperm from the tail of epididymides of rats, and this effect is consistent with those presented by Tanyildizi [26], who reported a similar effect of gossypol on sperm production in cattle. The effect of gossypol was reversed by the concomitant treatment of animals with vitamin E, indicating that the damage may be caused by its oxidizing activity. A similar effect was observed by Velasquez-Pereira et al. [27] when they evaluated the effect of vitamin E on sperm production in cattle. As a consequence of the effect of gossypol on sperm production of rats, we observed a decrease in fertility of the animals as measured by the smaller number and weight of offspring. Based on these observations, the effect of gossypol and vitamin E on the cellular antioxidant system was evaluated to determine the mechanisms involved.

Reduced glutathione (GSH) can be considered one of the most important agents of the cellular antioxidant defense system, protecting the cell against damage from exposure to oxidizing agents [28]. According to Halliwell and Gutteridge [29], physiologically, the body can defend itself against reactive oxygen species (ROS) using its reserves of antioxidants including reduced glutathione (GSH), nicotinamide adenine dinucleotide phosphate in the reduced form (NADPH); and using enzymes such as superoxide dismutase (SOD), catalase, glutathione peroxidase (GPx) and glutathione reductase (GR). An imbalance between the formation and removal of free radicals in the body, due to the reduction of endogenous antioxidants or the increased generation of oxidizing species, generates a pro-oxidant condition known as oxidative stress.

The treatment with 5 mg/kg BW gossypol induced a decrease in GSH and an increase in GSSG concentration in the rat testis homogenate. These changes in the redox cycle of glutathione are not consistent with those founded by El-Sharaky et al. [13], who evaluated the effect of gossypol in rats that received doses of 5, 10 and 20 mg/kg BW i.p. and observed increases in GSH and reductions in GSSG concentration. However, the results obtained in the present study are consistent with those found by Carvalho et al. [30] who observed a significant decrease in the level of GSH in liver homogenate of rats treated with 5 mg/kg BW gossypol compared to control animals, with a consequent increase in the concentration of GSSG. In addition, according to the results of the present study, vitamin E was able to protect against the oxidative effect of gossypol on glutathione.

The NAD system operates as an electron and H^+^ acceptor in the oxidation of organic substrates and the NADP system functions as a donor of reducing equivalents for biosynthetic processes. The ratio of [oxidized metabolite]/[reduced metabolite] reflects the redox state of pyridine nucleotides in the ratio NAD^+^/NADH and NADP^+^/NADPH. Furthermore, the pyridine nucleotides are a source of reducing equivalents necessary to remove endogenous and exogenous oxygen free radicals as well as the main reducing power for the reconstitution of the glutathione reductase/peroxidase enzyme system [31]. Treatment with gossypol also promoted the oxidation of NAD(P)H indicating a possible action on the activity of antioxidant enzymes, while vitamin E protected against this effect.

The results of this study showed an increase in the activity of glutathione peroxidase (GPx) and glutathione reductase (GR) in the testis homogenate of gossypol-treated animals compared with the control group. These data are in agreement with Bender et al. [32] who reported that gossypol can induce the formation of hydroperoxide (H_2_O_2_), which is highly toxic to cells and thus causes an increase in the activity of the GPx enzyme responsible for the conversion of H_2_O_2_ in H_2_O at the expense of reducing equivalents from GSH, which is in turn regenerated by the GR with the consequent oxidation of NAD(P)H.

According to Fornés et al. [23] and Peyster et al. [33], gossypol stimulates the generation of lipid peroxides, and these, in turn, promote damage to cell membranes. These effects are consistently illustrated in this study, since there was an increase in the formation of MDA in the testis homogenate of animals treated with gossypol, indicating the occurrence of lipid peroxidation. A similar result was reported by Carvalho et al. [30] when evaluating the effects of gossypol on rat livers. Treatment with vitamin E protected against this effect in the testes, which can be attributed to the fact that vitamin E is the major antioxidant in cell membranes, acting on polyunsaturated fatty acids and preventing their oxidation [34]. It is important to note that vitamin E reduces lipid peroxyl radicals to hydroperoxides, which require GPx activity in order to be converted into non-reactive molecules [35], explaining why vitamin E does not prevent the increase in GPx activity in the rats treated with gossypol.

Since gossypol is described as an uncoupler of mitochondrial respiration [4] it would be expected to reduce the intracellular ATP concentration. As expected, this effect was observed in the group treated with gossypol and not observed in the group treated with gossypol and vitamin E, showing that vitamin E protected against the toxicological effect of gossypol. Ueno [36] reported that gossypol decreases the production and the use of ATP and that this decrease in ATP production is directly related to the decrease in the sperm motility rate. In in vitro studies conducted by Wichmann et al. [37] using sperm incubated with gossypol, a dramatic drop in the production of ATP was also observed while ATP levels were unchanged in the control group. Moreover, reducing the concentration of ATP in the testes may have contributed to the decrease in number of spermatozoa observed in the present study, since energy is required in the process of spermatogenesis [38].

In conclusion, the mechanism of toxicity of gossypol on rat testis consists in the induction of oxidative stress and a reduction in ATP synthesis, leading to damage to cell membranes and reduced sperm production, resulting in a decline in fertility. Treatment of animals with vitamin E was shown to be effective in preventing oxidative damage caused by gossypol. Thus, the use of vitamin E can be suggested as a palliative measure in animals subjected to poisoning by gossypol.
